# Intestinal Failure in a Neonate: A Surgical Emergency and Medical Catastrophe

**DOI:** 10.7759/cureus.16890

**Published:** 2021-08-04

**Authors:** Pankaj Kumar Mohanty, Mohammad Zakiulla, Tapas Kumar Som, Bikasha Bihari Tripathy, Manoj Kumar Mohanty

**Affiliations:** 1 Neonatology, All India Institute of Medical Sciences, Bhubaneswar, Bhubaneswar, IND; 2 Pediatric Surgery, All India Institute of Medical Sciences, Bhubaneswar, Bhubaneswar, IND

**Keywords:** preterm, short gut, intensive care, parenteral nutrition, septic shock

## Abstract

In this case report, we present a female neonate referred to us, born to a primigravida mother at 39 weeks, who cried after birth, did not require any resuscitation, had a birth weight of 2.9 kg and developed abdominal distension and bilious vomiting on Day 1 of life. Ultrasound abdomen and X-ray imaging were suggestive of midgut volvulus with malrotation. The emergency explorative laparotomy revealed the small bowel to be gangrenous in extensive areas, and 10 cm of the small intestine was successfully preserved. The baby was admitted to the NICU and required three months of total parenteral nutrition. In between, the baby was managed successfully for sepsis, septic shock, diarrhea, and dehydration, Later, she was discharged, and is currently being followed up. At the first follow-up, the baby was noted to be gaining weight and has developed no complications to date.

## Introduction

Small bowel length in full-term neonates is approximately 200-300 cm and the colon is approximately 40 cm. The length usually doubles in the third trimester. The aetiologies of small bowel syndrome include necrotizing enterocolitis (NEC) (35%), meconium ileus and its complications (20%), gastroschisis (12.5%), intestinal atresia (10%), and volvulus (10%) [1]. Enteral autonomy can be best achieved with a 15 cm length of small intestine with ileocecal valve (ICV) and 40 cm without ICV [2,3]. Functioning bowel length is very vital for survival. The ultrashort small gut is defined as a gut length of 10 cm or less than 10% of the expected length of the small bowel [2]. Short bowel syndrome is a spectrum of malnutrition resulting from small bowel length, jejunal ileal atresia, and midgut volvulus. The reduction of functional small bowel length compromises the anatomy causing the physiological breakdown, which affects gastrointestinal absorptive function. Long-term recovery of these children often is usually normal, but there is a 10% to 15% incidence of neurologic impairment [4,5]. The mortality with SBS with intestinal failure is generally associated with the development of intestinal failure associated liver disease (IFALD) and sepsis. This case highlights that the attainment of enteral autonomy is feasible even with the occurrence of ultrashort gut.

## Case presentation

**Figure 1:**
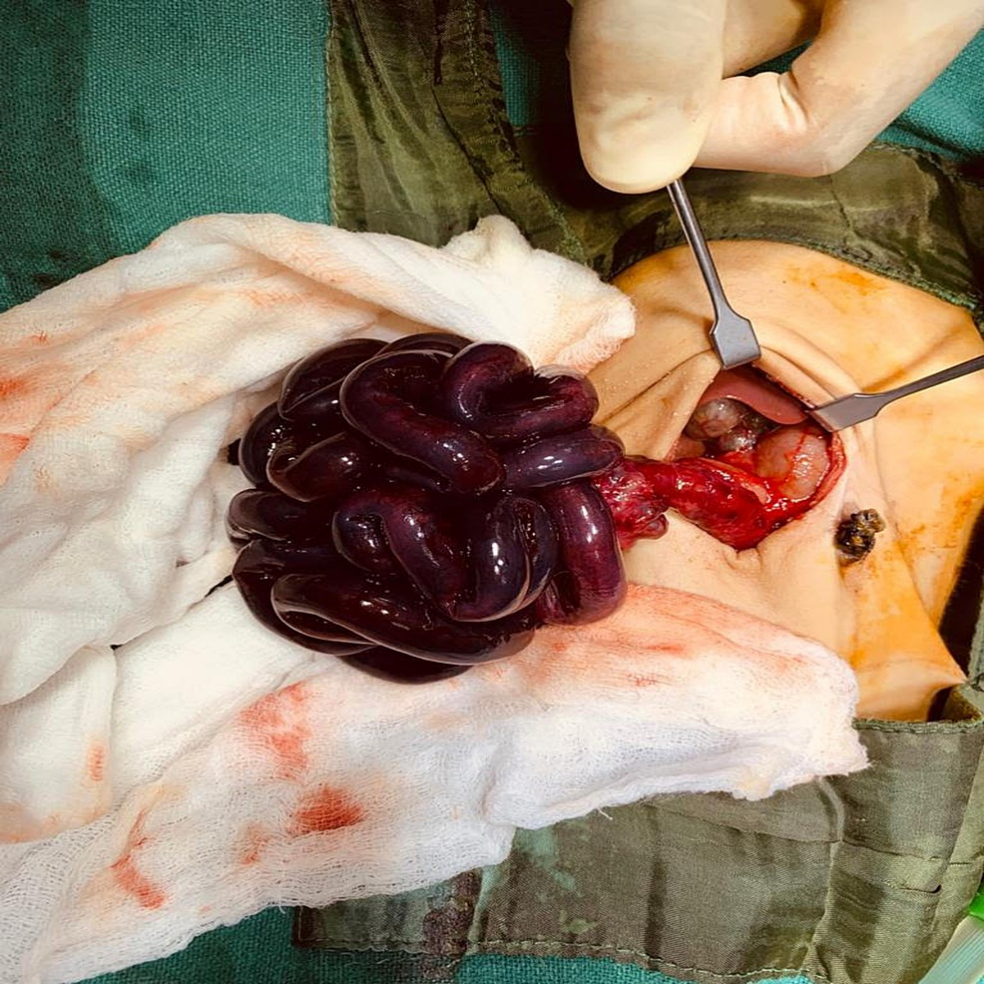
Mal-rotated bowel with volvulus and black gangrenous bowel 3-4 cm distal to deudeno-jejunal flexure up to 5 cm proximal to the ileocecal junction

**Figure 2:**
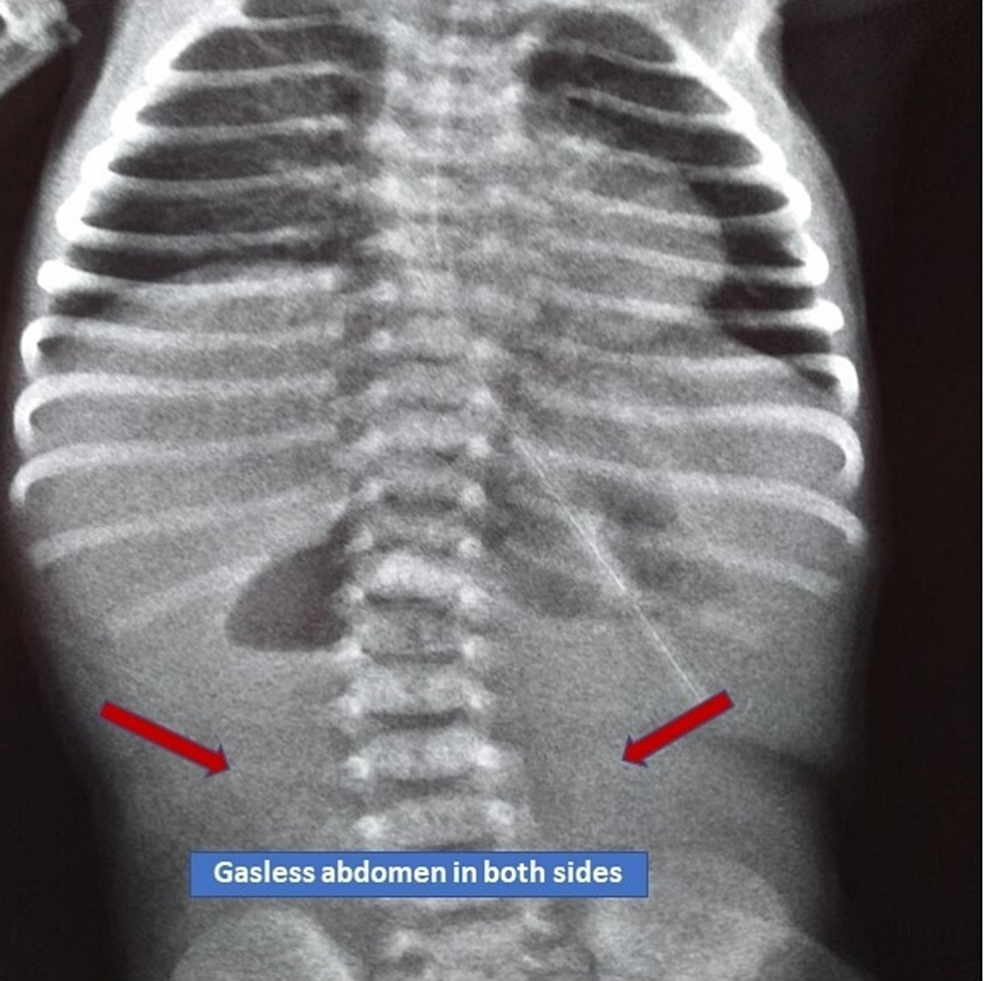
X-ray of abdomen PA view showing paucity of gas abdomen Paucity of gas abdomen is a sign of high small intestinal obstruction.

An outborn female baby was referred to a tertiary care hospital on Day 3 of life for abdominal distension and bilious vomiting since Day 1 of life. At admission, parameters were a temperature of 36.4⁰C, oxygenation in room air SPo2 94%, perfusion CRT< 3sec, and sugar at 93 mg%. The baby was hemodynamically stable, normothermic, euglycemic. Antenatally mother was booked and immunized in a peripheral hospital. Mother was 20 years of age, married for the last four years, and was seronegative as well as negative for HbsAg, HCV, and HIV. Perinatal history revealed a female baby born to a primigravida mother at 39 weeks of gestation through nonconsanguineous union by spontaneous vaginal delivery; the liquor was clear, and the baby didn't require any resuscitation. The baby developed several episodes of bilious vomiting and was kept nil by mouth and was on intravenous fluid. The baby had passed meconium and urine. After being admitted to NICU, routine investigations were conducted. The X-ray abdomen (Figure 1) and ultrasound abdomen were done, which was suggestive of midgut volvulus with malrotation. The baby had a deranged coagulation profile, for which he received fresh frozen plasma and platelet. Exploration laparotomy was done on the same day, and intraoperative findings revealed mal-rotated bowel with volvulus and black gangrenous bowel 3-4 cm distal to deudeno-jejunal flexure up to 5 cm proximal to the ileocecal junction (Figure 2). The entire gangrenous bowel was excised, the root of the existing bowel widened, Ladd's bands divided, and the end-to-end anastomosis of jejunum to ileum was done. The baby remained hemodynamically stable in the intraoperative period, and blood loss was minimal. The baby was shifted to NICU for postoperative and subsequent management. The baby remained on ventilatory support and total parenteral nutrition was started through the peripherally inserted central line. The baby received 8 mg/kg/min glucose, 3 gm/kg/day protein, and 3 gm/kg/day lipid, along with micronutrients like calcium, magnesium, and multivitamin. The baby developed multiple episodes of fever spike on Days 34 and 45 of life. The peripherally inserted central line (PICC) was changed at specific intervals. The blood culture and urine for fungal hyphae were sent. The culture report showed the growth of methicillin-resistant staphylococcus aureus (MSSA), for which sensitive antibiotics were added. The lumbar puncture was performed to rule out meningitis. The cerebrospinal fluid analysis was normal.

Minimal orogastric feed started on Day 20 of life. Feeds were gradually increased, and total parenteral nutrition (TPN) tapered and stopped once the baby reached 120ml/kg feeds with a calorie of 110 kcal/kg. The baby reached full feed on Day 84 of life. TPN was stopped, and direct breastfeed also started from Day 90 of life. To increase the calorie content for hydrolyzed casein dominant semi-elemental diet (Allimentum). The baby developed multiple episodes of acute watery diarrhea with no signs of dehydration which was managed with oral rehydration salts, zinc, and loperamide. The baby received a blood transfusion for anemia.

The baby remained hemodynamically stable and took direct feeds. Mother was actively involved in the care of the baby. The neurological examination at the time of discharge was normal. Parents have been explained in detail regarding follow-up care and the need for future intestinal lengthening procedures or intestinal transplantation in case the baby suffers from growth failure and other complications.

Anthropometric parameters of the index case

The anthropometric parameters (Table 1) showed that there was initial weight loss which was attributed to the primary illness and sepsis. The weight gain started from Day 14, but it did not follow the percentile due to other illnesses that the baby suffered, i.e., diarrhea and cholestasis. The baby was discharged on Day 105 of life 10 with the weight of 3030 g (< 3rd percentile), length 60.5 cm (50th to 97th percentile), and head circumference of 37 cm (< 3rd percentile). The failure to thrive was attributed to the hospital illness with diarrhea and infection that the baby had during hospital admission. The baby was showing consistent weight gain. At the time of discharge, the baby was healthy, hemodynamically stable, and breastfeeding well.

**Table 1:** Anthropometric parameters of the index case

	Day 3 (Admission)	Day 14	Day 30	Day 45	Day 60	Day 90	Day 105
Weight (g)	2375	2230	2445	2550	2715	2885	3030
Length (cm)	48	-	51	-	55	-	60.5
Head circumference (cm)	31.5	31.5	33	34.5	35.5	36	37

## Discussion

The management lines of the ultrashort gut should cover four crucial aspects. The first is effective management of fluid and electrolytes, and the second and most crucial part is improving nutrition through parenteral nutrition with the addition of micronutrients. The third phase is the introduction of enteral nutrition with essential amino acids, micronutrients., vitamins and minerals. The fourth phase is the introduction of semisolid foods to the infant that should consist of high protein, low to moderate fat, which should be introduced before carbohydrates [6].

Specific micronutrients like arginine and citrulline are found to reduce intestinal permeability in animal models. Citrulline also helps in intestinal adaptation. A low level of serum citrulline concentrations (a nonprotein amino acid produced by intestinal mucosa) has been proposed to predict permanent versus transient intestinal (less than 15 micromol/L) [7].

Omega-3 fatty acids are also shown to help in the adaptation of the small intestine and colon. In animal models, fish oil is seen to diarrhea and fecal fat excretion. Most of the studies of omega-3 fatty acid supplementation in humans are observational and limited to the pediatric population. Parenteral administration of lipids derived from fish oil (rich in omega-3 fatty acids) may reduce serum bilirubin levels and intestinal failure associated with liver disease. The SMOF (soya oil, medium-chain triglycerides, olive oil, and fish oil) lipid, which contains omega-3 fatty acids, was used to manage our index case [8].

Another micronutrient glutamine also shown to reverse intestinal Studies in humans have shown that enteral glutamine supplementation provides modest benefit in body weight and fluid and electrolyte balance. We couldn't use glutamine in nutrients due to the unavailability of the product [9].

The perioperative surgical decision regarding resection of segment and preservation of ileocecal valve and colon plays a critical role in the long-term outcome of these patients. Infants with a short gut and >10 cm small intestine usually gain intestinal autonomy. In our index case, the length of the small intestine was less than 10 cm (ultra-short). The growth failure was managed with long-term TPN. Intralipid should be replaced with SMOF lipid, which improves outcomes in patients with parenteral nutrition-associated liver injury [10]. We added multivitamins, calcium, and magnesium to the TPN. The essential micronutrients like zinc, phosphorous iron, and vitamin D were added with enteral nutrition. Other essential micronutrients couldn't be added in TPN as those were not available. The parenteral nutrition was stopped by 90 days as the baby showed weight gain with breast milk and casein formula. Breast milk is the best for such babies as it contains glutamine, different growth factors, and immunoglobulin A (IgA), which promotes intestinal adaptation and system. Our index case was fed with the mother's own milk, which was started in low volume initially (minimal enteral nutrition). Hydrolyzed casein dominant semi-elemental diet (Allimentum) was also added to increase the caloric content and to improve tolerance. The complications such as sepsis, cholestasis, diarrhea were managed successfully, and the baby was discharged with full enteral feeds. As per telephonic communication from the parents, the baby stayed 20 days at home without any complications. At the first follow-up, the baby was feeding properly and showing weight gain. 

## Conclusions

This case highlights the attainment of enteral autonomy is possible in controlled circumstances. The prevention of sepsis, early management of complications, tactically optimizing nutrition, and avoiding growth failure is essential to successful management in such neonates. Every case is different, and each case should be weighted by its clinical presentation and pathology inside. The aim of the treating clinical is to attain enteral autonomy early and avoid growth failure and other complications. Following up with these babies is very vital; ideally, they should reside within easy reach of the hospital, and any decision to send the baby away or to any higher health establishment should be taken with utmost precautions. Additionally, transportation should be handled with the help of a well-equipped medical team. 
